# An Optimized RT-qPCR Protocol for Comprehensive Analysis of microRNAs and mRNAs in *Mus musculus* Brain Tissues

**DOI:** 10.3390/ncrna12030020

**Published:** 2026-06-11

**Authors:** Maria V. Lukashevich, Margarita M. Rudenok, Suzanna A. Partevian, Maria I. Shadrina, Petr A. Slominsky, Anelya Kh. Alieva

**Affiliations:** National Research Centre “Kurchatov Institute”, 1 Akademika Kurchatova Square, Moscow 123182, Russia; margaritamrudenok@gmail.com (M.M.R.); s.partev@yandex.ru (S.A.P.); maria.i.shadrina@yandex.ru (M.I.S.); paslominsky@bk.ru (P.A.S.); anelja.a@gmail.com (A.K.A.)

**Keywords:** microRNA, expression analysis, method, stem-loop, real-time PCR, total RNA, TaqMan, stem-loop primers, SLP, protocol optimization

## Abstract

**Background/Objectives:** MicroRNAs are key post-transcriptional regulators involved in various diseases. Despite its status as the gold standard, real-time RT-PCR faces challenges arising from high sequence homology among closely related microRNAs and the substantial biomaterial required to enrich small RNA fractions. This study aimed to develop an optimized protocol for simultaneous analysis of microRNA and mRNA expression from a single total RNA sample using mouse (*Mus musculus*) brain tissue, avoiding dependence on pre-designed commercial assay panels. **Methods:** We optimized a real-time RT-PCR workflow enabling simultaneous analysis of mature microRNAs and mRNAs from a single total RNA sample. Modifications include a redesigned universal reverse primer, LNA-modified TaqMan probes, and omission of the 65 °C denaturation step during reverse transcription. The method was validated for five microRNAs in mouse brain tissue. **Results:** The assay showed high specificity, discriminating closely related *miR-125a-5p* and *miR-125b-5p* with a ΔCt difference of 6.7 ± 1.2 cycles. Co-analysis with Oligo(dT)_18_ and Random hexamer primers did not interfere with microRNA detection. **Conclusions:** The developed approach enables reliable detection of closely related microRNAs and parallel analysis of different RNA types, which is particularly important for studying regulatory networks when working with limited amounts of biomaterial. This protocol provides a complementary, accessible option for targeted studies in resource-limited settings or for non-cataloged miRNA targets.

## 1. Introduction

MicroRNAs (miRNAs) are a class of small non-coding RNAs approximately 18–25 nucleotides (nt) in length that play a crucial role in the post-transcriptional regulation of gene expression [1]. MiRNAs are involved in the control of proliferation, differentiation, and apoptosis and are implicated in the pathogenesis of numerous diseases, including oncological [2], neurodegenerative [3], and cardiovascular [4] conditions. Understanding the functions and regulatory networks linking miRNAs to their target genes can help identify promising expression biomarkers for more accurate and comprehensive diagnosis and treatment of diseases. In this regard, the precise determination of miRNA expression levels in a specific cell or tissue type is a crucial parameter for characterizing their role in both physiological and pathological conditions.

Currently, a wide range of methods are used to analyze miRNA expression, such as reverse transcription and real-time polymerase chain reaction (RT-PCR), microarray hybridization, next-generation sequencing (NGS), digital PCR, and others [5]. Among these, real-time RT-PCR is recognized as the “gold standard” for quantitative analysis, offering high sensitivity, accuracy, reproducibility, and an optimal cost-effectiveness ratio [6,7]. However, the small size of miRNAs (~22 nt), their low concentration in cells and extracellular fluids, as well as high homology within miRNA families, create significant methodological challenges for their detection and quantification [8,9].

To address the issue of small target size in real-time RT-PCR analysis of miRNAs, it has been proposed to use stem-loop primers (SLPs) during RT primer selection, which hybridize to the 3′-end of mature miRNA. This effectively extends the cDNA template for further analysis by real-time PCR [10].

There are several approaches to detecting amplification products. The most widely available method involves the use of intercalating dyes, such as SYBR Green I. However, this approach carries an increased risk of detecting nonspecific amplification products [11,12]. More specific methods involve the use of hydrolysis probes (e.g., TaqMan), which bind only to the complementary target sequence within the amplicon [13]. The use of an SLP and a specific TaqMan-probe in the analysis of mature miRNA expression minimizes the risk of detecting non-specific amplification products [10]. Currently, studies modifying this method do not account for the possibility of using total RNA for the parallel analysis of miRNA expression and the expression of protein-coding genes [14,15,16]. However, this imposes significant limitations on expression studies when working with hard-to-obtain and unique samples. Established commercial platforms have addressed integrated miRNA and mRNA analysis through specialized workflows. Thermo Fisher’s TaqMan MicroRNA Assays, particularly when combined with the Advanced miRNA cDNA Synthesis Kit, support combined detection by pooling stem-loop RT primers for miRNA alongside random primers for mRNA, frequently followed by preamplification and detection on TaqMan Array Cards [17]. However, these systems require proprietary reagents, pre-designed assay panels, or dedicated infrastructure that may be unavailable in some research settings.

The present protocol was developed to provide a fully customizable, cost-effective alternative using independently designed SLPs and LNA-modified hydrolysis probes synthesized by standard oligonucleotide providers [18]. This approach is intended for applications requiring analysis of miRNAs not covered by commercial catalogs, non-model species, or targeted regulatory studies where flexibility in primer design outweighs the benefits of integrated commercial panels.

The aim of this study was to develop and validate an optimized real-time RT-PCR protocol for the simultaneous analysis of miRNA and mRNA expression in a single total RNA sample. In this study, protocols were developed for the selection of primers for RT, as well as primers and TaqMan probes for real-time PCR for miRNAs. This allows for the effective comprehensive analysis of the expression of both miRNAs and protein-coding genes in cDNA samples derived from total RNA without small RNA enrichment. The effectiveness of the method has been demonstrated using samples of the frontal cortex of *Mus musculus* mice. This choice is due to the fact that mice are one of the most important and widely used model organisms in biomedical research [19,20].

## 2. Results

### 2.1. Design of Primer and TaqMan Probe Systems

Specific systems for the detection of miRNAs by real-time RT-PCR were designed based on the classic protocol by Chen et al. [10]. A universal SLP sequence was used for RT, and specificity to each target miRNA was ensured by a unique 6-nucleotide 3′-end of the primer, complementary to the 3′-end of the corresponding miRNA (Table 1).

**Figure 1:**
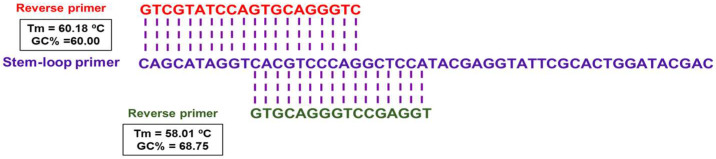
The following characteristics are exhibited by reverse primers when utilized in real-time PCR experiments. The SLP sequence is displayed in purple; the reverse primer sequence proposed in this study is displayed in red; and the reverse primer sequence from Chen et al. [10] is highlighted in green.

Two key modifications to the protocol for designing primers and TaqMan probes are proposed. The initial modification pertains to the selection of a sequence for a universal reverse primer for real-time PCR, a process that facilitated the optimization of its characteristics. This involved the improvement of the GC content and the reduction of the Gibbs free energy change for secondary structures (∆G) (Figure 1). An analysis was conducted using the Primer-BLAST program (version 2.5.0) [21,22], which demonstrated that the modified reverse primer exhibited increased specificity and a reduced risk of off-target product formation in comparison with the original version.

The second modification involved the use of LNA (Locked Nucleic Acid) nucleotides in the composition of TaqMan probes to achieve the required annealing temperature and increase the affinity of short TaqMan probes to DNA targets [23,24].

When selecting primers and TaqMan probes for real-time PCR, a unified set of criteria was utilized, which facilitates the concurrent analysis of miRNA and protein-coding gene expression in a single cDNA sample:

1. It is imperative to ensure that the guanine-cytosine (GC) content of the primers does not exceed 60%.

2. The annealing temperature of the forward and reverse primers must be 61 ± 1 °C.

3. It is imperative that the difference between the annealing temperatures of the forward and reverse primers is less than 1 °C.

4. The Gibbs free energy change (ΔG) for self-dimers, cross-dimers, and hairpin structures formed by these primers must not exceed 9 kcal/mol in absolute value, according to the “PrimersList tool for analyzing primer sequences” [25].

5. The annealing temperature of the TaqMan probe with LNA links must be 71 ± 3 °C (10 °C higher than that of the reverse and forward primers).

6. During the process of amplification, it is imperative that the primers and probe do not generate off-target products that are shorter than 450–500 base pairs (bp), as established by Primer-BLAST [21,22]. The experimental verification of annealing and primer specificity (not including the probe) was conducted utilising conventional PCR and electrophoresis in a 2% agarose gel.

**Figure 2:**
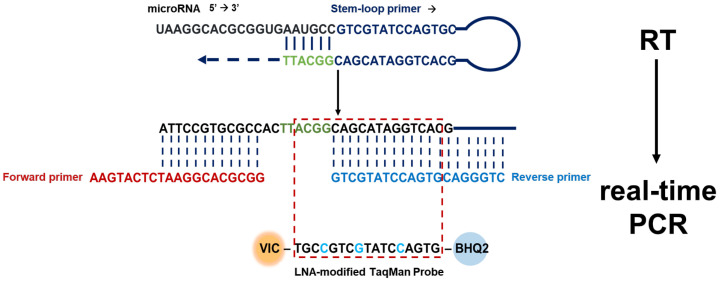
Schematic diagram illustrates the primer and TaqMan probe system for *miR-124-3p*. The sequence of the SLP is represented in blue, whilst the nucleotides of the SLP that are complementary to the miRNA are shown in light green. The forward primer sequence is represented in red, and the reverse primer sequence in blue. LNA linkers are highlighted in light blue, VIC is a fluorescent dye, and BHQ2 is a fluorescence quencher.

The following flowchart illustrates the mechanism of specific detection and discrimination of off-target products in the optimized real-time RT-PCR system (see Figure 2 for an example with the *miR-124-3p*).

Consequently, highly specific systems (SLPs for RT-PCR, primers and TaqMan probes for real-time PCR) were successfully selected for the detection of miRNAs *miR-124-3p*, *miR-125a-5p*, *miR-125b-5p*, *miR-135b-5p*, and *miR-205-5p*. The primer and probe systems that have been selected for real-time RT-PCR are presented in Table 2.

### 2.2. Optimization of Reverse Transcription Reaction Conditions

In the initial phase, RT was conducted in accordance with Protocol 1 (described in detail in the Section 4) and real-time PCR was performed to verify the presence of amplification products utilising the selected primer and TaqMan probe systems for the *Aars* and *miR-124-3p*.

**Figure 3:**
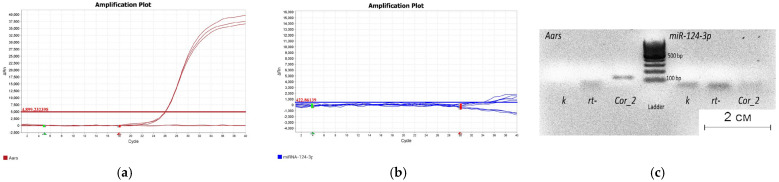
Real-time PCR accumulation curves according to Protocol 1: (**a**)—for the *Aars*; (**b**)—for *miR-124-3p*. (**c**) The electropherogram of the amplification products. k—negative control, rt-—RT control, and Cor_2—cDNA sample.

Consequently, reproducible amplification curves were obtained for the *Aars* (Ct values were 28.0 ± 0.3) in three replicates (Figure 3a) and a single band of the target size upon GE (Figure 3c), indicating successful detection of the specific product.

Conversely, for *miR-124-3p*, the accumulation curves remained below the detection threshold (Figure 3b), while GE revealed the presence of a small amount of amplification products less than 100 bp in length (Figure 3c), which likely corresponds to primer dimers or nonspecific products. This phenomenon has been linked to the absence of specialized SLP.

**Figure 4:**
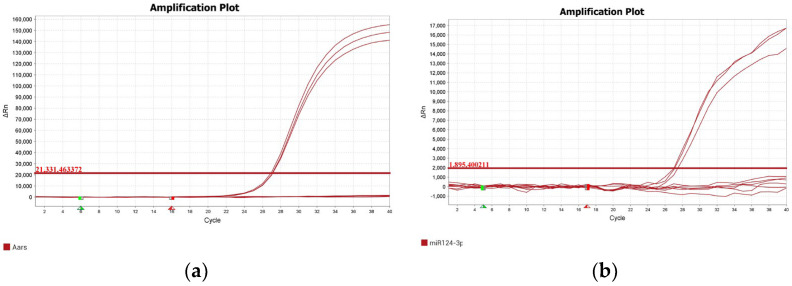
Real-time PCR amplification curves according to Protocol 2 with an SLP-mix incubation step at 65 °C (Temperature Regime 2.1); (**a**)—for the *Aars*; (**b**)—for *miR-124-3p*.

**Figure 5:**
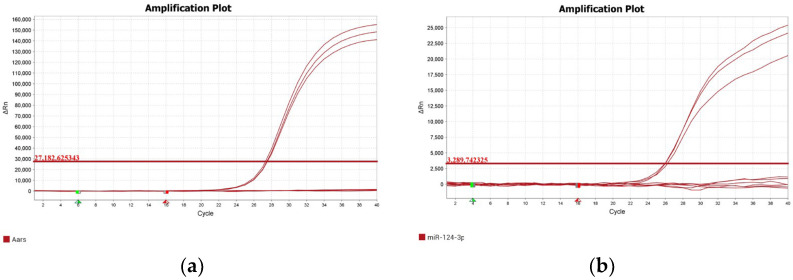
Real-time PCR amplification curves according to Protocol 2 without the SLP-mix incubation step at 65 °C (Temperature regime 2.2); (**a**)—for the *Aars*; (**b**)—for *miR-124-3p*.

In the second stage, RT-PCR was performed by adding specific SLP to the reaction mixture in accordance with Protocol 2 (described in detail in the Section 4). Two temperature profiles were used (see Section 4 for the full list of steps). Briefly, profile 2.1 included RNA denaturation at 65 °C, primer hybridization at 25 °C, cDNA synthesis at 42 °C, enzyme inactivation at 70 °C, and ice cooling; profile 2.2 started directly with cDNA synthesis without the initial denaturation step. The results of real-time RT-PCR are presented in Figure 4 and Figure 5.

As demonstrated in Figure 4 and Figure 5, the incorporation of an SLP is a prerequisite for the detection of *miR-124-3p*, as substantiated by the characteristic exponential accumulation curves (see Figure 4b and Figure 5b).

However, the efficiency of amplification was found to be contingent on the RT temperature profile employed. Under temperature profile 2.1, Ct values were 28.0 ± 0.5 for *Aars* and 27.0 ± 0.4 for *miR-124-3p*. Under temperature profile 2.2, the Ct values were one cycle lower, at 27.0 ± 0.4 and 26.0 ± 0.3, respectively. The utilization of temperature profile 2.2 yielded elevated levels of normalized fluorescence values (ΔRn) in comparison to the application of temperature profile 2.1 (see Figure 3 and Figure 4, respectively). In addition to quantitative indicators, the shape of the accumulation curves differed: when using temperature regime 2.2, they were characterized by a more stable and smooth increase in the signal until reaching the exponential phase (Figure 5).

Consequently, the absence of preheating (Temperature Profile 2.2) in the RT process resulted in enhanced cDNA synthesis efficiency and superior performance in subsequent real-time PCR when compared to the protocol involving denaturation at 65 °C.

**Figure 6:**
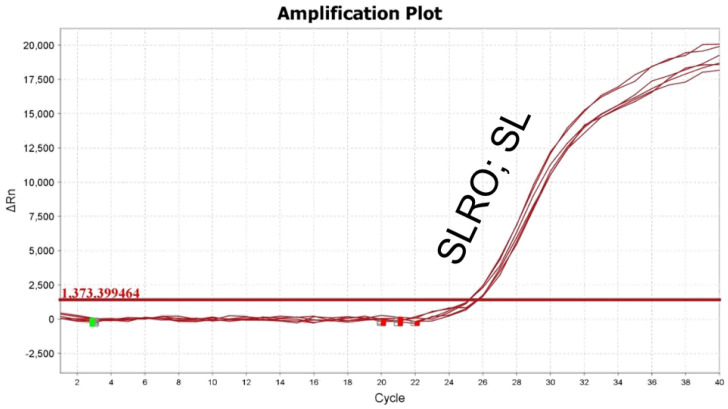
Real-time PCR amplification curves according to Protocol 2 for *miR-124-3p*. SL—sample with the addition of the *miR-124-3p* SLP, without Random Hexamer and Oligo(dT)_18_ primers during RT; SLRO—with the addition of the *miR-124-3p* SLP, Random Hexamer, and Oligo(dT)_18_ primers during RT.

In the subsequent stage of the investigation, the impact of incorporating primers for the synthesis of cDNA from protein-coding genes (Random Hexamer and Oligo(dT)_18_) within the RT reaction mixture on the detection of miRNA was examined (Figure 6). To this end, real-time RT-PCR was performed for *miR-124-3p* on two cDNA samples. In this case, Random Hexamer and Oligo(dT)_18_ primers, as well as an SLP specific for *miR-124-3p* according to Protocol 2, were added to the RT reaction mixture for the first sample (SLRO), while for the second sample (SL), only the SL primer specific for *miR-124-3p* was added.

As demonstrated in Figure 6, the accumulation curves for miRNA were found to be indistinguishable in the presence and absence of Random Hexamer and Oligo(dT)_18_ primers. This observation suggests that the efficiency and specificity of miRNA amplification are not contingent on the presence of these components. Statistical analysis confirmed equivalence between conditions: mean Ct for *miR-124-3p* was 25,8 ± 0.4 (SL, *n* = 3 technical replicates) versus 26.3 ± 0.5 (SLRO, *n* = 3 technical replicates), with a mean difference of 0.5 cycles (*p* = 0.25).

Therefore, the data demonstrate that SLPs facilitate specific cDNA synthesis using miRNA as a template without functionally competing with primers for mRNA from protein-coding genes, thereby enabling their combined use in a single RT reaction.

### 2.3. Validation of the Primer and TaqMan Probe Systems

**Figure 7:**
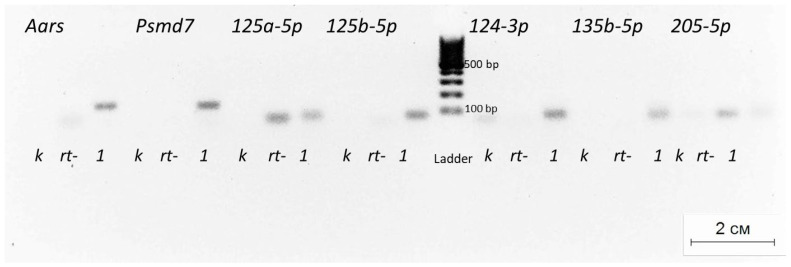
Electropherogram of real-time PCR products. k—negative control, rt-—RT reaction control, and 1—a sample of frontal cerebral cortex.

The specificity of the selected primer and TaqMan probe systems was confirmed by real-time PCR followed by agarose gel electrophoresis (Figure 7). As demonstrated in Figure 7, specific amplification products of the anticipated size were obtained for all miRNAs examined.

The validation of the selected TaqMan “primer–probe” systems for the analysis of the expression of *miR-124-3p*, *miR-125a-5p*, *miR-125b-5p*, *miR-135b-5p*, and *miR-205-5p* was performed using a sequential two-fold dilution of the cDNA template during real-time PCR. All validations were performed using Protocol 2.2. The *Aars* and *Psmd7* genes were utilized as internal controls [26]. The criterion for successful validation was the fulfilment of the condition |a| < 0.1 [27]. In addition, standard MIQE-compliant metrics were calculated: amplification efficiency (Efficiency (*E*) = (10^(−1/slope)^ − 1) × 100%) and R^2^ values for all calibration curves. All assays showed E between 92% and 107% and R^2^ ≥ 0.98, meeting accepted real-time PCR validation standards [28] (Table 3).

### 2.4. Accuracy and Specificity Verification of the Method Under Study for Detecting Closely Related miRNAs with Minimal Differences in Nucleotide Sequences

In order to analyze the specificity of the proposed protocol, primer and TaqMan probe systems were utilized for the miRNAs *miR-125a-5p* and *miR-125b-5p*. These miRNAs exhibit minor sequence variations (see Table 4).

Firstly, RT was performed on four total RNA samples (technical repetitions of the same biological sample) using various combinations of SL primers specific for *miR-125a-5p* and *miR-125b-5p*, as well as SLPs specific for the five miRNAs under study (see Table 5 for details).

**Figure 8:**
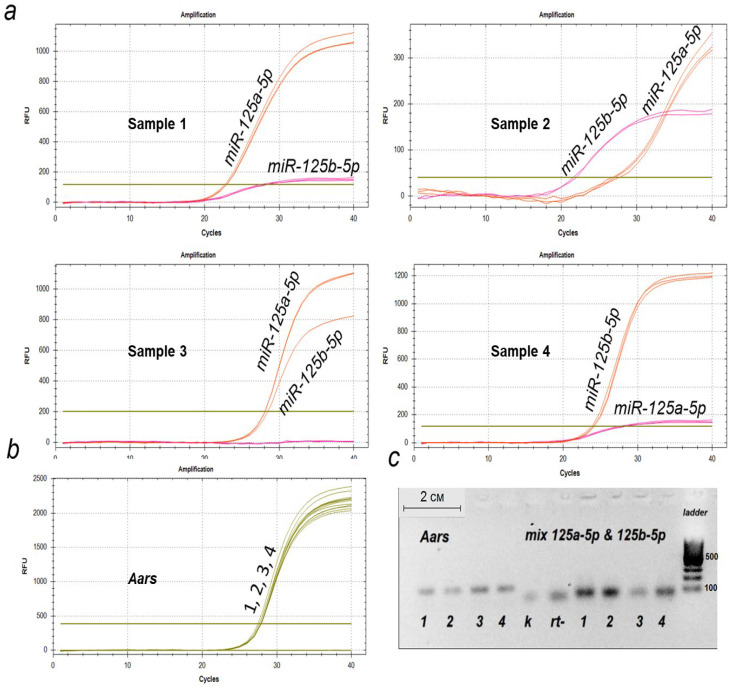
Results of real-time RT-PCR. (**a**) The real-time PCR accumulation curves for *miR-125a-5p* and *miR-125b-5p* are shown in pink and orange lines, respectively. (**b**) Green lines indicate the accumulation curves for *Aars*. (**c**) The electropherogram of the amplification products is shown below. “mix *miR-125a-5p* & *miR-125b-5p*”—PCR products from a single-tube duplex containing *miR-125a-5p* and *miR-125b-5p* primer sets, k denotes negative control, whilst rt-, 1 to 4 represent cDNA samples (see Table 5). The GeneRuler DNA Ladder size marker is indicated by “ladder”.

Subsequently, real-time PCR was performed on the obtained cDNA samples for *miR-125a-5p* and *miR-125b-5p*. Two fluorescent dyes with different maximum absorption wavelengths, VIC (538 nm) and Cy5 (645 nm), were utilized for the *miR-125a-5p* TaqMan probe and the *miR-125b-5p* TaqMan probe, respectively. The accumulation curves and the GE are shown in Figure 8, confirming the specificity of the amplification process.

In samples 1 and 2, obtained using SLPs specific for *miR-125a-5p* and *miR-125b-5p*, respectively, clear differences in real-time PCR results are observed. As demonstrated in Figure 8a, during the amplification of sample 1 for *miR-125a-5p*, the threshold Ct cycle was 23.0 ± 0.3. Concurrently, for *miR-125b-5p*, the Ct value was 29.5 ± 0.7. Conversely, sample 2 exhibited an inverse trend, with Ct values of 22.4 ± 0.4 and 28.2 ± 0.6 observed for *miR-125b-5p* and *miR-125a-5p*, respectively. In accordance with the tenets of quantitative PCR, this finding signifies that the amplification efficiency of the target sequence is approximately 64 times greater than that of the non-target, homologous sequence. This constitutes direct evidence of the high specificity of the developed systems. In addition to the threshold cycles, discrepancies are also evident in the maximum fluorescence level (ΔRn) between the specific and nonspecific signals, which indirectly reinforces the selectivity of these primer systems.

In addition, in sample 3, which comprised cDNA synthesized in the presence of SL-primers for both miRNAs, both targets were reliably detected by the corresponding systems. However, *miR-125a-5p* exhibited a measurable Ct shift (≈4 cycles) relative to single-plex detection in sample 1, indicating primer competition. Furthermore, in sample 4, prepared using a complete mixture of five different SLPs, the discrimination between the two closely related miRNAs was maintained. Consequently, the selected systems attain a sufficiently elevated level of specificity for the target sequence, thereby enabling effective discrimination between non-target cDNA.

However, the result shown in Figure 8a for sample 4 may be due to competitive inhibition under conditions where various primers are present in excess in a single RT-PCR reaction mixture. This could potentially affect the efficiency of RT-PCR for specific targets, or to the formation of secondary structures that hinder hybridization. This emphasizes the necessity of pre-optimizing the composition and quantity of primers in the RT-PCR mixture when analyzing multiple targets simultaneously. In order to achieve maximum efficiency and reproducibility within this protocol, it is recommended that critical or low-abundance targets, such as *miR-125a-5p*, be analyzed in separate RT-PCR reactions or as part of minimal, carefully selected primer mixtures of no more than three to four SLPs. Rather than analyzing these targets in a single reaction with the entire miRNA panel for expression analysis, this approach is to be preferred.

## 3. Discussion

Accumulated evidence indicates that miRNAs play significant roles in the pathogenesis of various diseases [29]. Accordingly, analyzing miRNA expression patterns is of considerable interest for developing methods for early diagnosis and targeted therapy of various pathologies. Nevertheless, contemporary analytical methodologies are subject to several limitations. For instance, microarray hybridization lacks sufficient specificity and offers a narrow dynamic range, while NGS and digital PCR require expensive equipment and labor-intensive protocols [5]. Real-time RT-PCR, provided that appropriate reaction conditions and detection methods are selected, remains one of the most reliable and effective approaches for analyzing miRNA expression.

In this study, we proposed a modification of a widely used protocol for primer design in real-time RT-PCR for the analysis of mature miRNA expression. This modification was based on the original protocol described in 2005 by Chen et al. [10]. The primary objective of this study was to adapt the method for use with total RNA, thereby enabling the parallel analysis of both miRNAs and RNA from protein-coding genes in a single sample. In this particular context, the conserved spike region of the SLP for RT was retained in its original state, as its sequence is already optimal for efficient miRNA extension. The specificity of the RT level was ensured by a unique 3′-end of the primer complementary to the target miRNA (see Table 1).

A pivotal modification entailed the alteration of the sequence of the universal reverse primer for real-time PCR. This enabled a substantial enhancement in its thermodynamic parameters, thereby augmenting its specificity. This observation was corroborated by analysis employing the Primer-BLAST program [21,22] (Figure 1). The utilization of a primer is known to ensure a lower level of nonspecific background and to facilitate more stable and efficient amplification.

The second significant modification was the transition from MGB labels, which were commonly used in commercial systems, to LNA modifications in TaqMan probes. It is evident that, due to the short maturation of miRNAs, the utilization of standard-sized probes is rendered unfeasible (25–30 nt). The LNA modification facilitated an increase in the Tm of the short oligonucleotide without an increase in its length, thereby enabling the creation of high-affinity probes that met all specified thermodynamic parameters [23,24].

We optimized the RT protocol. The protocol utilized for the analysis of mRNA expression is predicated on the employment of random hexamer and Oligo(dT)_18_ primers for reverse transcription (RT). It is evident from Figure 3 that this protocol is not capable of detecting short RNA molecules. This is due to the fact that short mature miRNAs are not able to function as templates for standard RT primers. The slight background signal observed in Figure 3b is likely attributable to the amplification of nonspecific products in the accumulation curves. The incorporation of specific SLP into the reaction mixture effectively resolved this issue, as demonstrated by the emergence of characteristic accumulation curves (Figure 4b and Figure 5b) and the formation of amplification products of the anticipated size (Figure 7).

However, it was found that preheating the reaction mixture, a step frequently recommended in RT-PCR protocols to reduce RNA secondary structures [14,15,16], had a negative impact on the efficiency of subsequent miRNA amplification (Figure 4). Conversely, the omission of this step and the execution of RT-PCR utilizing temperature profile 2.2 yielded stable and reproducible results (Figure 5b). As demonstrated in the publication “Small Non-Coding RNAs. Methods and Protocols” [30], it has been established that pre-incubation of the mixture of RNA template and primers for the RT reaction can lead to changes in the secondary structure of SLPs. It is therefore important to note that this step is appropriate only for linear RT primers. It can be hypothesized that the correct configuration of SLPs (a hairpin structure with a protruding end complementary to a specific miRNA) is necessary for specific hybridization with the target miRNA. In the context of SLPs utilized for the amplification of multiple miRNAs, this structural configuration functions to impede their mutual hybridization, thereby enabling the concurrent generation of cDNA for multiple miRNAs.

A further significant finding is the demonstration that miRNA and mRNA can be analyzed simultaneously within a single RT reaction. The experimental demonstration was conducted to ascertain the impact of the simultaneous presence of primers for mRNA (Random Hexamer and Oligo(dT)_18_) and SLPs in the reaction mixture on the efficiency and specificity of miRNA amplification. The results obtained from this experiment are illustrated in Figure 6. This approach has been shown to engender substantial savings in biological material, reagents, and time, a factor that is of particular significance when dealing with unique or limited samples.

Consequently, we have developed detection systems for five miRNAs based on SL primers and LNA-containing TaqMan probes, which have been validated (Table 2 and Table 3). This combination ensured high system specificity, as demonstrated by the closely related miRNAs *miR-125a-5p* and *miR-125b-5p*. The primer and TaqMan probe systems for these miRNAs demonstrated high specificity towards the target cDNA sequence (Figure 8).

However, it is important to note that the result shown in Figure 8a for sample 4 may be due to competitive inhibition under conditions where various primers are present in excess in a single RT-PCR reaction mixture. This could potentially affect the efficiency of RT-PCR for specific targets, or to the formation of secondary structures that hinder hybridization. This emphasizes the necessity of pre-optimizing the composition and quantity of primers in the RT-PCR mixture when analyzing multiple targets simultaneously. In order to achieve maximum efficiency and reproducibility within this protocol, it is recommended that critical or low-abundance targets, such as *miR-125a-5p*, be analyzed in separate RT-PCR reactions or as part of minimal, carefully selected primer mixtures. Rather than analyzing these targets in a single reaction with the entire miRNA panel for expression analysis, this approach is to be preferred.

The capacity to concurrently analyze miRNAs and their prospective mRNA targets from a solitary total RNA sample is of considerable biological significance. This approach eliminates the variability that arises when these two classes of RNA are analyzed separately.

The recent literature underscores the expanding clinical utility of miRNA quantitative PCR across diverse sample types and disease contexts. In liquid biopsy applications, stem-loop RT-PCR has been successfully deployed for plasma-based cancer biomarker panels, serum miRNA profiling in ovarian cancer, and urinary extracellular vesicle analysis for early-stage renal cell carcinoma [31].

These biofluid applications impose stringent sensitivity requirements, as circulating miRNAs are present at low concentrations and can be degraded by nucleases. LNA-modified probes have proven particularly valuable in this context because they increase thermal stability and mismatch discrimination without requiring proprietary MGB chemistries, enabling shorter probe designs that maintain adequate melting temperatures [18]. Beyond liquid biopsies, FFPE tissue archives represent an enormous untapped resource for retrospective biomarker validation. Recent investigations have demonstrated that carefully optimized RT-PCR workflows can yield reproducible miRNA expression data from FFPE blocks, highlighting the need for methods that tolerate partially degraded RNA and the value of simultaneous miRNA and mRNA analysis to capture intact regulatory networks in archival material. The omission of a 65 °C preheating step in our RT protocol may be particularly advantageous for FFPE applications, as excessive heat exposure can exacerbate RNA fragmentation and compromise the structural integrity of SLPs [30].

However, we emphasize that these represent theoretical advantages only. FFPE RNA is heavily cross-linked and fragmented, which may block SLP hybridization and reduce mRNA RT efficiency beyond the levels observed in fresh-frozen tissue. Liquid biopsy specimens contain RNases and inhibitors that were not present in our brain tissue lysates. Therefore, adaptation of this protocol to FFPE or biofluid samples would require dedicated validation including DNase pretreatment, inhibitor removal, and empirical optimization of primer concentrations for each specimen type.

However, it is essential to acknowledge the limitations of this protocol with appropriate rigor. First, the validation scope is restricted to five miRNAs and two reference genes in mouse frontal cortex tissue. RNA concentration was quantified by Qubit fluorometry (mean 46.61 ng/μL) but conventional purity metrics (e.g., A260/A280, A260/A230, or RIN) were not assessed. Extension to other species, tissues, or partially degraded samples—including the FFPE and biofluid specimens discussed above—requires dedicated validation.

Second, primer competition imposes a strict limit on multiplexing: our data show that combining five SLPs in one RT reaction completely suppresses low-abundance targets, and even two SLPs cause measurable Ct shifts. We quantified this competition effect in Figure 8, where *miR-125a-5p* exhibited a ΔCt of +4 cycles when co-incubated with one additional SLP relative to single-plex RT, and complete signal loss when four additional SLPs were present. For quantitative biomarker studies, we recommend no more than three to four SLPs per RT reaction, with critical or low-abundance targets analyzed in separate reactions.

Third, we have not performed head-to-head benchmarking against commercial integrated systems such as TaqMan Array Cards [17] or miRCURY LNA panels, as these require proprietary infrastructure not utilized in this study. Users should evaluate whether the flexibility of custom design outweighs the standardized quality control and extensive validation datasets provided by commercial alternatives.

Finally, although the method reduces material requirements for combined analysis, it does not reduce the number of reactions needed for comprehensive miRNA profiling. We have therefore added a quantitative scalability analysis: for four or fewer miRNAs plus reference genes, a single RT reaction suffices and material savings are maximal; for five to ten miRNAs [32], two to three reactions are needed, yielding moderate savings compared to separate small RNA and total RNA preparations; for more than ten targets, the protocol becomes impractical and commercial multiplexed solutions or NGS are preferable. The primary advantage thus lies in targeted regulatory studies examining specific miRNA–mRNA pairs in limited or unique samples, not in global miRNome screening.

## 4. Materials and Methods

### 4.1. Tissue Preparation

The study’s subjects were male mice of the C57Bl/6 strain (*n* = 5). The utilization of laboratory animals was conducted in accordance with the Guide for the Care and Use of Laboratory Animals [33], the 3Rs principles (replacement, reduction, and refinement) [34], and the requirements of the Local Ethics Committee on Biomedical Research at the National Research Centre “Kurchatov Institute” (Protocol No. 1pr of the Local Ethics Committee of the NRC “Kurchatov Institute” dated 16 January 2024). The animals were euthanized by cervical dislocation. Immediately following the dissection, tissue samples were obtained from the frontal lobes of the cerebral cortex. The samples were then frozen using dry ice and stored at −70 °C until further use.

### 4.2. Isolation of Total RNA from Mouse Brain Tissue

The RNA was isolated from 20 ± 5 mg of frontal cerebral cortex using a method based on the Trizol reagent (Tri reagent; Molecular Research Center, Inc., Cincinnati, OH, USA). Phase separation was achieved by the addition of chloroform (0.2 volumes relative to the volume of Trizol), followed by centrifugation for 15 min at a temperature of +4 °C and at a rotational speed of 12,000 rpm [35]. Subsequently, the aqueous phase containing RNA was collected, and ethanol was added in a 1:1 ratio. Then total RNA was isolated using the Direct-zol™ RNA MiniPrep kit (Zymo Research, Irvine, CA, USA), in accordance with the manufacturer’s instructions. The RNA concentration was measured using the Qubit^®^ RNA HS Assay Kit on a Qubit 3.0 fluorometer according to the manufacturer’s instructions (Thermo Fisher Scientific, Waltham, MA, USA). The mean RNA concentration was 46.61 ± 17.64 ng/μL.

### 4.3. Primers and TaqMan Probes Selection for Real-Time PCR

Mature miRNA sequences were obtained from the MirBase database (Release 22.1) [36]. The design of mature miRNA SLPs for RT and mature miRNA primers with TaqMan probes for real-time PCR was performed based on the method described by Chen et al. [10]. Sequences of reference genes primers with TaqMan probes for real-time PCR were described by Alieva et al. [26]. The specificity of the primer and TaqMan probe system was verified using the Primer-BLAST program (Primer3 version 2.5.0) [21] and the RefSeq database [22]. Primers were synthesized by Eurogen (Moscow, Russia), and TaqMan probes were synthesized by DNA-Synthesis (Moscow, Russia).

### 4.4. Reverse Transcription Reaction

The RT reaction was performed on a T3 Thermocycler (Biometra, Göttingen, Germany) using two protocols. Protocol 1 is the standard RevertAid First Strand cDNA Synthesis protocol (Thermo Fisher Scientific, Waltham, MA, USA) for generating cDNA from mRNA templates of protein-coding genes. The distinguishing feature of Protocol 2 is the incorporation of a combination of SLPs (an equimolar amount of each SLP) for all miRNAs tested in this study and modified concentrations of Oligo(dT)_18_ and Random Hexamer primers. The composition of the reaction mixtures for Protocols 1 and 2 is presented in Table 6.

Protocol 2 had two temperature conditions (2.1 and 2.2), which are shown in Table 7.

### 4.5. Real-Time PCR Assay, Employing the TaqMan System

For the real-time PCR assay, the template was constituted by cDNA samples obtained from the RT reaction. Real-time PCR was performed on a QuantStudio 3 amplifier (Applied Biosystems, Foster City, MA, USA). Prior to the reaction, cDNA was diluted in an aqueous solution of yeast tRNA (100 ng/μL) to a concentration of 0.02 ng/μL [37]. The composition of the 30 μL reaction mixture comprised 5 μL of cDNA (0.02 ng/μL), 3 μL of PCR buffer (×10, Sintol, Moscow, Russia), 3 μL of 25 mM MgCl_2_, 10 nM of primers (Eurogen, Moscow, Russia), 2.5 nM of a probe (DNA-Synthesis, Moscow, Russia), 200 μM of each dNTP, and 1 U of Taq DNA polymerase (Sintol, Moscow, Russia) [38]. In order to account for variations in sample quality and reverse transcription reaction efficiency, each sample was analyzed in triplicate.

### 4.6. Validation of the Selected Primer and TaqMan Probe Sets

The primer and TaqMan probe sets were validated using a serial two-fold dilution of the template during real-time PCR. For each dilution, Ct values were determined for both the target miRNAs and the reference genes (*Aars* and *Psmd7*) [26]. To construct calibration curves, ΔCt values were used, calculated as the difference between the Ct of the target miRNA and the average of the Ct values of the reference genes for each dilution. Calibration curves were plotted in the log[N]/ΔCt coordinate system. The resulting relationships were described by the linear regression equation y = ax + b, where the coefficient |a| reflects the change in fluorescence signal per order of magnitude of concentration, and the value $\frac{1}{a}$ characterizes the change in DNA quantity per amplification cycle. The criterion for successful validation was the fulfillment of the condition |a| < 0.1 [27]. Additionally, amplification efficiency (E = 10^(−1/slope)^ − 1) and R^2^ were calculated for each assay to ensure MIQE compliance [28].

### 4.7. Agarose Gel Electrophoresis (GE)

A gel with a 2% agarose concentration in 1× TAE buffer and the GeneRuler 100 bp DNA Ladder (Thermo Fisher Scientific, Waltham, MA, USA). The criterion for amplification specificity was the presence of a single band corresponding to the calculated length of the PCR product, determined during the selection of primer sets and TaqMan probes.

### 4.8. Data Analysis

The Ct values and amplification curves were obtained using QuantStudio 3 software (Applied Biosystems, Foster City, CA, USA) and Bio-Rad CFX Manager (Bio-Rad Laboratories, Inc., Hercules, CA, USA). The specificity of the manually selected primers was further verified using the Primer-BLAST program (Primer3 version 2.5.0) [21] and the RefSeq database [22]. The analysis was conducted utilising the MS Excel 2019 software package (Microsoft, Washington, DC, USA). Statistical comparisons were performed using paired *t*-tests and one-way ANOVA (Supplementary Table S1, Figure S1) where appropriate; *p* < 0.05 was considered significant.

## 5. Conclusions

In summary, an optimized real-time RT-PCR protocol has been developed and validated. The aim of this method is to perform simultaneous expression analysis of miRNAs and mRNAs from a single total RNA sample, which is advantageous for studying intact regulatory networks (non-coding RNAs and their target genes) and is applicable for working with limited and hard-to-obtain samples. The protocol was validated for five miRNAs and two reference genes in *Mus musculus* frontal cortex tissue. It is positioned as a flexible, accessible alternative to proprietary commercial systems for laboratories requiring custom miRNA target selection or operating outside commercial catalog coverage. A detailed step-by-step bench protocol is provided in Supplementary Materials (Supplementary Material Document S1) to facilitate direct implementation.

## Figures and Tables

**Table 1:** Nucleotide sequences of SLPs for RT-PCR targeting the miRNAs *miR-124-3p*, *miR-125a-5p*, *miR-125b-5p*, *miR-205-5p*, and *miR-135b-5p*.

MiRNA	Nucleotide Sequence (5′ to 3′)
*miR-124-3p*	GTCGTATCCAGTGCAGGGTCCGAGGTATTCGCACTGGATACGAC**GGCATT**
*miR-125a-5p*	GTCGTATCCAGTGCAGGGTCCGAGGTATTCGCACTGGATACGAC**TCACAG**
*miR-125b-5p*	GTCGTATCCAGTGCAGGGTCCGAGGTATTCGCACTGGATACGAC**TCACAA**
*miR-205-5p*	GTCGTATCCAGTGCAGGGTCCGAGGTATTCGCACTGGATACGAC**CAGACT**
*miR-135b-5p*	GTCGTATCCAGTGCAGGGTCCGAGGTATTCGCACTGGATACGAC**TCACAT**

Bold/underlined text indicates the unique 6-nucleotide sequence at the 3′ end of the primer, which is complementary to the 3′ end of the corresponding miRNA.

**Table 2:** Sequences of selected primers and TaqMan probes for miRNAs in real-time PCR.

miRNA		Nucleotide Sequence (5′ to 3′)	T_m_ (°C)	|ΔG| ^1^ (kcal/mol)	GC (%)
Reverse primer (universal)		GTCGTATCCAGTGCAGGGTC	60.2	None	60.0
*miR-124-3p*	Forward primer	AAGTACTCTAAGGCACGCGG	59.8	5.3	55.0
	TaqMan Probe (**LNA**)	*-TGC**C**GTC**G**TATC**C**AGTG-***	71	None	41.2
*miR-125a-5p*	Forward primer	AGCGTTCCCTGAGACCCTT	60.5	6.3	57.9
	TaqMan Probe (**LNA**)	*-TACGA**C**TCA**C**AGGTTAAAGG-***	71	1.9	30.0
*miR-125b-5p*	Forward primer	CAGCATCCCTGAGACCCTAA	60.8	1.7	55.0
	TaqMan Probe (**LNA**)	*-/**-GATA**C**GA**C**TC**A**CAA**C**TT-***	71	6.7	23.5
*miR-205-5p*	Forward primer	CGCGGCATCCTTCATTCCA	60.8	None	57.9
	TaqMan Probe (**LNA**)	*-AC**G**A**C**CAG**A**CT**C**AGA-***	71	1.6	33.3
*miR-135b-5p*	Forward primer	CCGCTCGTATGGCTTTTCATTC	60.3	None	50.0
	TaqMan Probe (**LNA**)	*-GTG**A**G**T**CGT**A**TCC**A**GTG-***	71	None	52.9

^1^ The maximum value of the Gibbs free energy, ΔG, for the formation of self- and cross-dimers between the primers and the probe is shown. The fluorescent dyes used are VIC (*) and Cy5 (**); the fluorescence quencher is BHQ2 (***); and the LNA links are shown in bold.

**Table 3:** Values obtained during validation of the selected systems for real-time PCR.

miRNA	Equation	Value |a|	R^2^	E
*miR-124-3p*	y = 0.0674x + 1.1754	0.0674	0.99	107.2%
*miR-125a-5p*	y = 0.0784x − 2.18	0.0784	0.98	107.1%
*miR-125b-5p*	y = −0.0195x − 0.4055	0.0195	0.99	105.1%
*miR-205-5p*	y = −0.0144x + 4.233	0.0144	0.99	92.0%
*miR-135b-5p*	y = 0.0773x + 3.6787	0.0773	0.99	103.8%

**Table 4:** Sequences of mature *miR-125a-5p* and *miR-125b-5p*.

miRNA	Nucleotide Sequence (5′ to 3′)
*miR-125a-5p*	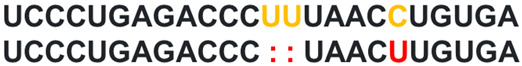
*miR-125b-5p*	

Different colors (red and yellow) represent sequence differences between mature microRNA.

**Table 5:** List of the cDNA samples used in experiment to detect closely related miRNAs.

No	Sample Description
1	cDNA sample with SLP for miR-125a-5p added
2	cDNA sample with SLP for miR-125b-5p added
3	cDNA sample with SLPs for miR-125a-5p and miR-125b-5p added
4	cDNA sample with the addition of SLPs for all five miRNAs under study
rt-	RT reaction control

**Table 6:** The composition of the RT reaction mixtures.

Reagent	Protocol 1	Protocol 2
	Quantity × 1 (μL)	
Buffer	4	
dNTPs	2	
SLPs	-	2.5
Oligo (dT)_18_/Random Hexamer primers	1/1	0.2/0.3
RevertAid Reverse Transcriptase	1	
RiboLock RNase Inhibitor	1	
Water	to a total volume of 20 μL	-
RNA	10	9

**Table 7:** Temperature conditions of the RT reaction for Protocol 2.

Stages of the Reverse Transcription Reaction	Temperature Profile 2.1	Temperature Profile 2.2
Denaturation of the RNA template and primer hybridization	65 °C—5 min	–
Specific primer-target hybridization and initiation of synthesis	25°C—5 min	
cDNA synthesis	42°C—1 h	
Thermal inactivation of reverse transcriptase	70°C—5 min	
cDNA stabilization	immediately transferred to ice	

## Data Availability

The raw data supporting the conclusions of this article will be made available by the authors on request.

## Supplementary Materials

The following supporting information can be downloaded at: https://www.mdpi.com/article/10.3390/ncrna12030020/s1, Figure S1: ∆Ct distribution across biological replicates in mice (Cortex), Table S1: Assessment of biological replicate homogeneity by one-way ANOVA and Kruskal–Wallis test., Document S1: Step-by-Step real-time RT-PCR Protocol for Simultaneous miRNA and mRNA Analysis from a Single Total RNA Sample.

## Abbreviations

The following abbreviations are used in this manuscript:

GE	gel electrophoresis
LNA	Locked Nucleic Acid
MGB	Minor Groove Binder
miRNA	microRNA
mRNA	messenger RNA
NGS	next-generation sequencing
nt	Nucleotide
PCR	polymerase chain reaction
RT	reverse transcription
SLP	stem-loop primer
TAE	Tris-acetate-EDTA
