# Supplementary material for: An Optimized RT-qPCR Protocol for Comprehensive Analysis of microRNAs and mRNAs in Mus musculus Brain Tissues
Source: Noncoding RNA. 2026 Jun 11;12(3):20. doi: 10.3390/ncrna12030020 (PMC13304521; doi:10.3390/ncrna12030020)
Supplement: Supplementary file 1 [file ncrna-12-00020-s001.zip › Suplementary Materials/Supplementary Material Document S1.pdf]

Step-by-Step real-rime RT-PCR Protocol for Simultaneous miRNA and mRNA Analysis from a Single Total RNA Sample

This protocol describes based on the classic protocol by Chen et al. [1] the complete workflow for simultaneous reverse transcription and real-time PCR analysis of mature microRNAs and protein-coding mRNAs from a single total RNA sample, using standard laboratory reagents and custom-synthesized oligonucleotides. The procedure is optimized for mouse brain tissue but can be adapted for other tissues with appropriate validation.

1. Reagents and Equipment

1.1. Reagents

- ✓ Tri Reagent (Molecular Research Center, Inc., Cincinnati, USA) or equivalent single-step RNA isolation reagent
- ✓ Chloroform, molecular grade
- ✓ Ethanol (96–100%), molecular grade
- ✓ Direct-zol RNA MiniPrep kit (Zymo Research Corp., California, USA) or equivalent column-based RNA purification system
- ✓ Qubit RNA Assay Kit (Thermo Fisher Scientific, Massachusetts, USA)
- ✓ NanoDrop spectrophotometer or equivalent UV-Vis system
- ✓ RevertAid First Strand cDNA Synthesis Kit (Thermo Fisher Scientific, Massachusetts, USA)
- ✓ DNase I, RNase-free (1 U/μL) (Thermo Fisher Scientific, Massachusetts, USA) or equivalent
- ✓ Stem-loop primers (SLPs) for target miRNAs, lyophilized to preserve its potency (Eurogen, Russia or equivalent) .
- ✓ Nuclease-free water
- ✓ Taq DNA polymerase (Sintol, Russia or equivalent) 5 U/μL
- ✓ 10× PCR Buffer (Sintol, Russia or equivalent)
- ✓ 25 mM $MgCl_2$
- ✓ dNTP Mix, 10 mM each (Eurogen, Russia or equivalent)
- ✓ Forward and reverse PCR primers, lyophilized to preserve its potency (Eurogen, Russia or equivalent).
- ✓ LNA-modified TaqMan hydrolysis probes, lyophilized to preserve its potency (DNA-Synthesis, Russia or equivalent)
- ✓ Yeast tRNA carrier, 100 ng/μL in nuclease-free water
- ✓ GeneRuler 100 bp DNA Ladder (Thermo Fisher Scientific, Massachusetts, USA)
- ✓ Agarose, molecular grade
- ✓ 1× TAE Buffer

1.2. Equipment

- ✓ Tissue homogenizer-grinder
- ✓ Refrigerated centrifuge (capable of 13,000 rpm at +4 °C)
- ✓ T3 Thermocycler or equivalent programmable thermal cycler
- ✓ QuantStudio 3 Real-Time PCR System (Applied Biosystems, California, USA) or equivalent
- ✓ Qubit 3.0 Fluorometer (Thermo Fisher Scientific, Massachusetts, USA)
- ✓ NanoDrop spectrophotometer
- ✓ Bioanalyzer 2100 (optional, for RIN assessment)
- ✓ Standard gel electrophoresis apparatus
- ✓ UV transilluminator or gel documentation system

1.3. Software and Online Resources

- **NCBI** (https://www.ncbi.nlm.nih.gov) — Public sequence database.
- **miRBase** (https://mirbase.org) — MicroRNA sequence and annotation database (Release 22.1).
- **Primer-BLAST** (https://www.ncbi.nlm.nih.gov/tools/primer-blast/) — Primer design tool; used to evaluate sequence properties (Tm, GC%) and assay specificity, including primer–primer and primer–probe interactions.
- **sRNAPrimerDB** (http://srnaprimerdb.com) — Primer design repository; used to select suitable assay systems and assess sequence characteristics, e.g., Gibbs free energy of dimer formation.
- **OligoCalc** (http://biotools.nubic.northwestern.edu/OligoCalc.html) — Oligonucleotide property calculator; used to estimate approximate melting temperatures and generate complementary sequences.
- **Multiple Primer Analyzer** (https://horizondiscovery.com/en/ordering-and-calculation-tools/multiple-primer-analyzer) — Tool for screening self- and cross-dimer formation among primers.
- **PrimersList Tool for Analyzing Primer Sequences** (https://primerdigital.com/tools/PrimerList.html) — More stringent algorithm for evaluating self- and cross-dimer formation among primers.

2. Steps

2.1. Step 1: Tissue Homogenization and Total RNA Isolation

1. Harvest tissue immediately after euthanasia. For the mouse frontal cortex, dissect rapidly and freeze in dry ice. Store at −70 °C until processing.
2. Place in a sterile tube containing 1 mL of Tri Reagent per 50 mg of tissue.
3. Homogenize tissue thoroughly until no visible particles remain. Incubate the homogenate at room temperature for 5 min to permit complete dissociation of nucleoprotein complexes.
4. Add a chloroform (1:5 ratio to the Tri Reagent). Cap the tube securely and shake vigorously for 30 s. Incubate at room temperature for 2–3 min.
5. Centrifuge at 12,000 rpm for 15 min at +4 °C. The mixture will separate into three phases: a lower red phenol-chloroform phase, an interphase, and a colorless upper aqueous phase containing RNA.
6. Carefully aspirate the upper aqueous phase (approximately 100–120 µL) without disturbing the interphase. Transfer to a fresh tube.
7. Add an equal volume of 96% ethanol (1:1 ratio) to the aqueous phase. Mix by pipetting for 10–15 s. Do not centrifuge.
8. Proceed immediately to column purification using column-based RNA purification kit according to the manufacturer's instructions.
9. Elute RNA in 30–50 µL of nuclease-free water.
10. Place purified RNA on ice and proceed immediately to DNase treatment according to the manufacturer's instructions, or store at −70 °C for up to 4 weeks.

2.2. Step 2: RNA Quantification and Quality Control

1. Measure RNA concentration using the Qubit RNA Assay Kit according to the manufacturer's instructions. Record concentration in ng/µL.
2. Assess purity by UV spectrophotometry (NanoDrop). Acceptable ratios: A260/A280 = 1.9–2.1; A260/A230 = 2.0–2.3. Ratios outside this range indicate protein or organic solvent contamination; repurify if necessary.
3. If available, assess RNA integrity using a Bioanalyzer 2100. RIN > 7.0 is recommended for optimal RT-qPCR performance. However,

this protocol has been validated with high-quality RNA from flash-frozen tissue without requiring RIN assessment.

4. Adjust all samples to the same concentration (e.g., 10 ng/µL) using nuclease-free water, or use equal volumes of stock RNA for RT reactions.

2.3 Step 3. Preparing for Primers design

To facilitate navigation through sequence records during primer design, create a color-coding legend to distinguish nucleotide sequences:

miRNA — all miRNA sequences are highlighted with a marker.
- Complementary strand — all sequences built as reverse complements of the preceding sequences.
- *Reverseprimer* — all sequences matching the reverse primer sequence.
- Tm-optimization nucleotides — nucleotide bases added to increase the annealing temperature.

Example (used hereafter):

MIMAT0000238 *miR-205-5p* (miRBase)

5′-UCCUUCAUUCCACCGGAGUCUG-*3′*

Navigate to sRNAPrimerDB, select 'Primer Bank' → 'miRNA', and fill in the required fields:

method (select Stem-loop RT-PCR), species, and the target miRNA from the list. If an assay system for the selected miRNA is present in the database, the following window will open.

Here, a specific stem-loop RT primer (RTprimer(specific)) can be retrieved; however, for convenience, we use the universal sequence of this primer optimized for melting temperature:

5′-GTCGTATCCAGTGCAGGGTCCGAGGTATTCGCACTGGATACGAC-3′

2.4 Step 4: Design the specific SLP for reverse transcription

As noted above, we use the universal stem-loop primer: 5′-GTCGTATCCAGTGCAGGGTCCGAGGTATTCGCACTGGATACGAC-3′.

Specificity is achieved by appending six nucleotides to the 3′ end of this sequence that are complementary to the 3′ end of the target miRNA (uracil is replaced by thymine):

5′-GTCGTATCCAGTGCAGGGTCCGAGGTATTCGCACTGGATACGACCAGACT-*3′*

2.5 Step 5: Design the forward and reverse primers for real-time PCR

Copy the first 12–17 nucleotides from the 5′ end of the mature miRNA sequence (uracil → thymine). The melting temperature should be approximately 61 °C. If necessary, add 3–7 nucleotides to the 5′ end to achieve the desired Tm.

*F: 5′-*CGCGGCA *TCCTTCATTCCA-3′ Tm = 60.82 °C*

For the reverse primer we use a universal sequence.

R: 5′-GTCGTATCCAGTGCAGGGTC-3′ Tm = 60.18 °C

2.6 Step 6: TaqMan probe design

The probe should be 12–17 nucleotides in length with a melting temperature of approximately 70 °C (accounting for LNA-modification). Thus, the probe sequence itself should have a Tm > 50 °C.

a) Identify the reverse primer sequence within the stem-loop structure according to the legend:

5′*-GTCGTATCCAGTGCAGGGTC*CGAGGTATTCGCACTGGATACGAC CAGACT *— 3′*

b) Append the sequence complementary to the miRNA at the 3′ end:

*5′-GTCGTATCCAGTGCAGGGTC*CGAGGTATTCGCACTGGATACGAC CAGACTCAGACTCCGGTGGAATGAAGGA *— 3′*

c) Build the reverse complement of the resulting sequence. Add the extra nucleotides from the 5′ end of the forward primer to the 5′ end of this complement. Then identify the forward primer sequence according to the legend:

5′— CGCGGCA*TCCTTCATTCCACCGGAGTCTGAGTCTG*GTCGTATCCAGTGCGAATACCTCG *GACCCTGCACTGGATACGAC —3′*

d) Remove the 3′ nucleotides of the reverse primer to obtain the top-strand sequence and its complementary bottom strand.

Next, mark the probe sequence on the bottom strand in bold. The probe must span the junction between the mature miRNA sequence and the stem-loop, without overlapping the forward primer.

For:

5′— CGCGGCA*TCCTTCATTCCA*CCGGAGTCTGAGTCTGGTCGTATCCAGTGCGAATACCTCG*—3′*

Rev:

5′— CGAGGTATTCGCACTGGAT**ACGACCAGACTCAGA**CTCCGG*TGGAATGAAGGA*TGCCGCG*—3′*

Probe:

5′—(VIC) ACGACCAGACTCAGA*-3′ (Quencher) Tm = 49.12 °C*

Then add LNA – modified bases to achieve the desired Tm. Do not place LNA at the very 3′ end, as this may inhibit Taq polymerase 5′→3′ exonuclease activity.

2.7 Step 7: Dimer check

Use the following online tools to screen for self- and cross-dimers among primers and probe:
- Multiple Primer Analyzer
- PrimersList Tool for Analyzing Primer Sequences

Use Primer-BLAST with the following databases:
- RefSeq mRNA, RefSeq Representative Genomes, etc.

The assay must not produce off-target products shorter than 500 bp. Melting temperatures for all sequences are also verified in Primer-BLAST.

Final Data (Example)

Stem-loop primer:
5′-GTCGTATCCAGTGCAGGGTCCGAGGTATTCGCACTGGATACGACTCACAG-3′

Name	Sequence (5′→3′)	Length	Tm (°C)	GC (%)
Forward primer	CGCGGCATCCTTCATTCCA	19	60.82	57.89
Reverse primer	GTCGTATCCAGTGCAGGGTC	20	60.18	60.00
Probe	ACGACCAGACTCAGA	17	49.12	53.33

Requirements for Primer and TaqMan Probe Design

1. It is imperative to ensure that the guanine-cytosine (GC) content of the primers does not exceed 60%.
2. The annealing temperature of the forward and reverse primers must be 61°C ± 1°C.
3. It is imperative that the difference between the annealing temperatures of the forward and reverse primers is less than 1°C.
4. The Gibbs free energy change (ΔG) for self-dimers, cross-dimers, and hairpin structures formed by these primers must not exceed 9 kcal/mol in absolute value, according to the "PrimersList tool for analyzing primer sequences" [2].
5. The annealing temperature of the TaqMan probe with LNA links must be 71 ± 3°C (10°C higher than that of the reverse and forward primers).
6. During the process of amplification, it is imperative that the primers and probe do not generate off-target products that are shorter than 450–500 base pairs (bp), as established by Primer-BLAST [3,4]. The experimental verification of

annealing and primer specificity (not including the probe) was conducted utilising conventional PCR and electrophoresis in a 2% agarose gel.

Otherwise, the primer–probe system will be inefficient.

2.8 Step 8: Reverse Transcription (Optimized Protocol 2.2)

Critical notes: Use only Temperature Profile 2.2 (no 65 °C preheating). Preheating destabilizes SLP structure and reduces miRNA detection efficiency. All reagents must be kept on ice during preparation.

1. Prepare the RT master mix on ice for the required number of reactions.
2. Per reaction (20 μL final volume):

5× Reaction Buffer: 4 μL

10 mM dNTP Mix: 2 μL

Stem-loop primer mix (3 μL each): 2.5 μL

If analyzing a single miRNA: 2.5 μL of the specific SLP

If analyzing multiple miRNAs: combine SLPs at 3 μL each, total volume 2.5 μL

Do not exceed 4 different SLPs per reaction to avoid competition-induced signal loss

Random Hexamer (100 μM): 0.3 μL

Oligo(dT)$_{18}$ (100 μM): 0.2 μL

RevertAid Reverse Transcriptase (200 U/μL): 1 μL

RiboLock RNase Inhibitor (20 U/μL): 1 μL

Nuclease-free water: to 11 μL (adjust based on RNA volume)

3. Add 11 μL of master mix to each PCR tube.
4. Add 9 μL of RNA sample (containing 10–100 ng total RNA) to each tube. Mix by gentle pipetting. Do not vortex.
5. Incubate the reaction mixture at 25 °C for 5 min to allow SLP hybridization to miRNA targets.
6. Synthesize cDNA at 42 °C for 60 min.
7. Inactivate reverse transcriptase at 70 °C for 5 min.
8. Cool immediately on ice for at least 2 min.
9. Store diluted cDNA at −20 °C or proceed directly to real-time PCR.

2.9 Step 9: Real-Time PCR Setup

1. Prepare the qPCR master mix on ice.

Per reaction (25 μL final volume):

10× PCR Buffer: 3 μL

25 mM $MgCl_2$: 3 μL (final 2.5 mM)

Forward primer: 1 μL (final 10 nM)

Reverse primer: 1 µL (final 10 nM)
LNA-modified TaqMan probe: 1 µL (final 2.5 nM)
mM dNTP Mix: 0.6 µL (final 200 µM each)
Taq DNA polymerase (5 U/µL): 0.2 µL (final 1 U)
Nuclease-free water: 12,8 µL

2. Prepare cDNA template (0.02 ng/µL) (5 µL final volume):
Nuclease-free water: 4 µL
Yeast tRNA: 1 µL
cDNA: 0,25 µL

3. Controls for each assay:
No-template control (NTC): 5 µL nuclease-free water instead of cDNA. Must yield Ct > 38 or undetermined.
RT-minus control (RT−): RNA sample processed through RT without reverse transcriptase enzyme. Must yield Ct > 35 or undetermined after DNase treatment.
Positive control (Mandatory): cDNA from a validated sample known to express the target miRNA.
4. Thermal cycling program:
Initial denaturation: 95 °C for 3 min
40 cycles of:
Denaturation: 95 °C for 15 s
Annealing/Extension: 60 °C for 30 s (fluorescence acquisition on FAM/VIC/Cy5 channel)
Melting curve analysis (optional but recommended):
95 °C for 15 s
60 °C for 1 min
Ramp to 95 °C at 0.05 °C/s with continuous fluorescence acquisition
5. Confirm single peak at expected Tm (~82–85 °C for specific products; primer-dimers appear at ~75–78 °C)

2.10 Step 10: Data Analysis and Normalization

1. Export Ct values from the real-time PCR instrument software. Exclude wells with failed NTC amplification (Ct < 38) or abnormal amplification curves.
2. Calculate the mean Ct from three technical replicates for each biological sample. Exclude individual replicates deviating by >0.5 cycles from the median.

3. Normalize miRNA expression to the geometric mean of two validated reference genes (*Aars* and *Psmd7*). Calculate ΔCt = Ct(target miRNA) − mean(Ct(reference genes)).
4. For relative quantification between samples, use the ΔΔCt method. Report fold change as 2^(−ΔΔCt).
5. Construct calibration curves by serial two-fold dilution of a pooled cDNA sample. Verify that $|a| < 0.1$ in the equation $y = ax + b$, where $y = \log_2(\text{dilution factor})$ and $x = \Delta Ct$. Additionally confirm that amplification efficiency ($E = 10^{(-1/slope)} - 1$) is between 90% and 110% and $R^2 \geq 0.98$.
6. For multiplexed miRNA panels, ensure identical SLP composition across all samples within an experiment. Do not compare absolute Ct values between samples prepared with different numbers or combinations of SLPs in the RT reaction, as primer competition causes systematic Ct shifts (competition coefficient C ≈ 0.6–0.9 cycles per additional SLP for low-abundance targets).

3. Troubleshooting Guide

Problem: No amplification in miRNA wells (Ct undetermined)

Verify that stem-loop primers were added to the RT reaction. Without SLP, mature miRNA cannot serve as RT template.

Check SLP stock concentration and storage. SLPs should be stored at −20 °C in aliquots; avoid repeated freeze-thaw cycles.

Confirm that the 25 °C hybridization step was performed before 42 °C cDNA synthesis.

Verify probe dye compatibility with your real-time PCR instrument.

Problem: High background in NTC (Ct < 38)

Check for cross-contamination during master mix preparation. Prepare NTC first, using fresh pipette tips.

Examine melting curve: broad low-Tm peak indicates primer-dimer formation. Reduce primer concentration or redesign primers.

Replace all reagent stocks; contamination may be present in water or buffer.

Problem: Positive signal in RT− control

Indicates genomic DNA contamination. Repeat DNase I treatment (Step 1) and verify with intron-spanning primers.

Ensure DNase was fully inactivated before RT; residual DNase will degrade cDNA during qPCR.

Problem: Sub-50 bp bands in gel but clean qPCR curves

These are stem-loop primer dimers formed during PCR cycling. They are invisible to TaqMan probes because the probe requires the miRNA-SLP junction. No action needed if NTC is clean in qPCR.

To reduce gel artifacts, load negative control lanes first with fresh tips.

Problem: ΔCt shift >1.5 cycles when multiplexing SLPs

Reduce the number of SLPs per RT reaction to ≤4.

Analyze low-abundance miRNAs in separate single-plex RT reactions.

Empirically titrate SLP concentrations: reduce high-abundance miRNA SLP concentration by 50% to balance competition.

Problem: Low amplification efficiency (E < 85% or E > 115%)

Check primer-dimer formation by melting curve analysis.

Verify primer concentrations (10 nM final in qPCR).

Test annealing temperature gradient (58–64 °C).

Recalculate efficiency using the correct slope from the linear regression of log(dilution) vs. Ct (not ΔCt).

4. Important Notes

RNA stability: Flash-freeze tissue immediately after dissection. Avoid storage at −20 °C; use −70 °C or colder.

Multiplexing limit: Do not combine more than 3–5 SLPs in a single RT reaction if quantitative comparison between samples is required. For exploratory screening, up to 6 SLPs may be used, but low-abundance targets will be suppressed.

Probe design: LNA-modified bases should be positioned at the 5′ and 3′ ends of the probe and at mismatch-sensitive internal positions. Do not place LNA at the very 3′ end, as this may inhibit Taq polymerase 5′→3′ exonuclease activity.

Reagent substitution: This protocol was validated with RevertAid reverse transcriptase and Sintol Taq polymerase. Substitution with other enzymes (e.g., SuperScript IV, Phusion polymerase) requires revalidation of reaction conditions and primer annealing temperatures.

Clinical sample adaptation: For FFPE or liquid biopsy samples, this protocol requires dedicated validation. FFPE RNA requires extended protease K digestion (20 min at 55 °C) before RT. Plasma/serum samples require miRNA enrichment or increased input volume due to low RNA yield.

5. Protocol Inquiries

For questions regarding this protocol, contact the corresponding author: lukashevich.mv@phystech.edu

References

1. Chen, C.; Ridzon, D.A.; Broomer, A.J.; Zhou, Z.; Lee, D.H.; Nguyen, J.T.; Barbisin, M.; Xu, N.L.; Mahuvakar, V.R.; Andersen, M.R.; et al. Real-time quantification of microRNAs by stem-loop RT-PCR. *Nucleic Acids Res* **2005**, *33*, e179, doi:10.1093/nar/gni178.
2. Kalendar, R. Comprehensive web-based platform for advanced PCR design, genotyping, synthetic biology, molecular diagnostics, and sequence analysis. *Molecular Therapy Nucleic Acids* **2025**, *36*, 102716, doi:https://doi.org/10.1016/j.omtn.2025.102716.
3. Ye, J.; Coulouris, G.; Zaretskaya, I.; Cutcutache, I.; Rozen, S.; Madden, T.L. Primer-BLAST: a tool to design target-specific primers for polymerase chain reaction. *BMC bioinformatics* **2012**, *13*, 134, doi:10.1186/1471-2105-13-134.
4. Sayers, E.W.; Beck, J.; Bolton, E.E.; Brister, J.R.; Chan, J.; Comeau, D.C.; Connor, R.; DiCuccio, M.; Farrell, C.M.; Feldgarden, M.; et al. Database resources of the National Center for Biotechnology Information. *Nucleic acids research* **2024**, *52*, D33-d43, doi:10.1093/nar/gkad1044.
