# Supplementary material for: An Optimized RT-qPCR Protocol for Comprehensive Analysis of microRNAs and mRNAs in Mus musculus Brain Tissues
Source: Noncoding RNA. 2026 Jun 11;12(3):20. doi: 10.3390/ncrna12030020 (PMC13304521; doi:10.3390/ncrna12030020)
Supplement: Supplementary file 1 [file ncrna-12-00020-s001.zip › Suplementary Materials/Supplementary Material Table S1 Figure S1.pdf]

Supplementary Material 1.

All qPCR measurements were performed in triplicate technical runs (3 PCR wells per cDNA sample). Biological replication comprised n = 5 male C57Bl/6 mice. Inter-mouse variability was assessed for each miRNA by one-way ANOVA (parametric) and Kruskal–Wallis test (non-parametric). p-values are reported uncorrected, with Bonferroni and FDR (Benjamini–Hochberg) corrections for multiple comparisons. Mean range and pooled standard deviation (SD) are shown for descriptive context. No significant inter-mouse differences were detected for any of the five miRNAs (all ANOVA $p > 0.05$).

Table S1: Assessment of biological replicate homogeneity by one-way ANOVA and Kruskal–Wallis 516 test

miRNA	ANOVA p	Bonferroni	FDR	Kruskal p	Mean range	Pooled SD
miR-124-3p	0.673	1.000	0.721	0.374	0.49	0.45
miR-125a-5p	0.060	0.301	0.220	0.126	0.33	0.09
miR-125b-5p	0.142	1.000	0.265	0.197	0.57	—
miR-135b-5p	0.088	1.000	0.220	0.156	0.95	0.38
miR-205-5p	0.245	1.000	0.334	0.225	0.68	0.40

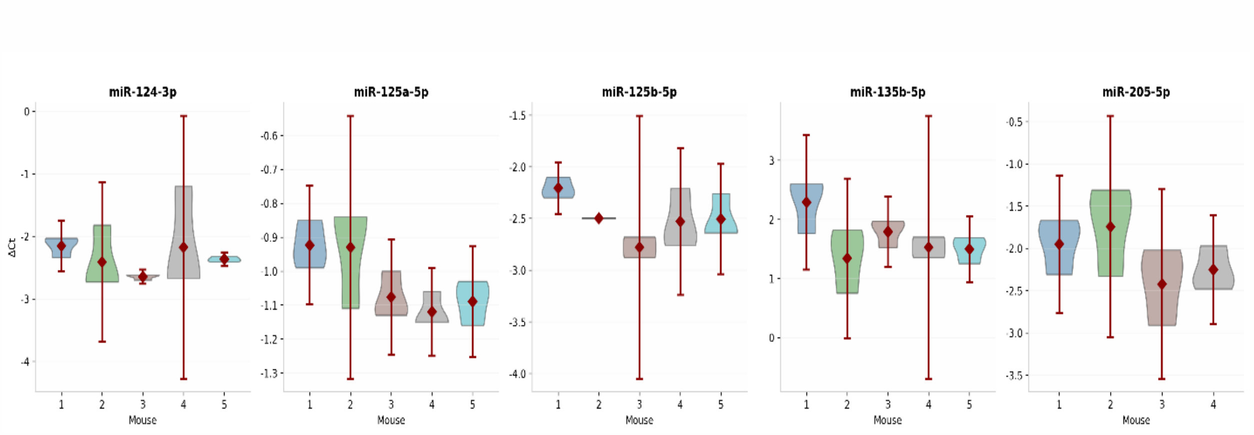

Figure S1: ΔCt distribution across biological replicates in mice (Cortex)
